# An *In Vitro* Evaluation of the Red Cell Damage and Hemocompatibility of Different Central Venous Catheters

**DOI:** 10.1155/2020/8750150

**Published:** 2020-04-14

**Authors:** Alojz Ihan, Stefan Grosek, David Stubljar

**Affiliations:** ^1^Institute of Microbiology and Immunology, Medical Faculty of Ljubljana, University of Ljubljana, Ljubljana, Slovenia; ^2^Department of Pediatric Surgery and Intensive Therapy, University Medical Centre Ljubljana, Ljubljana, Slovenia; ^3^Department of Research and Development, In-Medico, Metlika, Slovenia

## Abstract

**Background:**

The aim of our study was to evaluate the damaging impact of characteristics of the central venous catheters (CVCs) on red blood cells.

**Methods:**

CVCs from three different manufacturers were analyzed, including the presence of coating, tunnel geometry, length, lumen diameter, and number of lumens with two respective flow rates (33 mL/min and 500 mL/min). Blood cell damage was defined by analyzing microparticle (MP) and hematologic analysis. MPs were isolated by ultracentrifugation of erythrocyte concentrate and analyzed on a flow cytometer.

**Results:**

Characteristics of catheters were not associated with blood cell damage at a low flow rate but showed an effect with a high flow rate. CVCs with a polyhexanide methacrylate coating have caused statistically less blood cell damage than noncoated CVCs. The length of lumens, diameter, and geometry of the tunnel showed no differences in blood cell damage. Meanwhile, the number of lumens was predicted to have a greater effect on the erythrocyte damage, which was revealed with the formation of MPs and hematological parameters. CVCs with five lumens caused significantly less damage to the blood cells than CVCs with a single lumen. Moreover, a high flow rate of 500 mL/min caused less damage to the blood cells than a low rate of 33 mL/min.

**Conclusion:**

Properties of CVCs are an important factor for quality patient care, especially when transfusing blood with high flow rates, as we want to provide a patient with high-quality blood with as few damaged cells as possible.

## 1. Introduction

Blood cells can become damaged if they encounter artificial surfaces such as plastic, metal, or glass [1] due to cavitation, collisions between the cells [2], and turbulent flows [3]. Therefore, exposure to the catheters might be a potential reason for blood cell damage. The quality of transfused blood taken for analyses or transfusion is important. Blood quality relies on the damage of red blood cells and when these cells burst or lyse, they extract free hemoglobin into plasma [4].

Although catheters improve the health of patients, their hydrophobic character of surface materials accelerates protein adsorption and cell adhesion, leading to various undesirable complications [5]. Hydrophobic surfaces adsorb more proteins than hydrophilic [6]. Thus, the most common complications are damage to circulating blood components, the formation of blood clots, activation of the complement system and other immune pathways [7], hemolysis, and release of hemoglobin into the plasma [3]. Protein adsorption and blood cell adhesion, as well as the formation of blood clots, can compromise the function of the devices, in such a way that they impede the blood flow [7].

When the blood is exposed to the device, first the protein layer begins to adsorb onto the artificial surface [7] and reaches a thickness of 2 to 10 nm [8]. Adsorption of blood plasma proteins is a rapid process that creates a biologically active surface within seconds and can interact with other blood mechanisms [9]. Meanwhile, when the adsorbed proteins return to the equilibrium, the adhesion of blood cells (platelets, erythrocytes, and leukocytes) to the protein layer begins [10]. The dynamics of protein adsorption is related to the type of protein and the chemical and physical properties of the surface [11]. Platelet adhesive receptors recognize adsorbed proteins on artificial surfaces, which can lead to platelet adhesion, propagation, and activation. Potential mediators for platelet adhesion are fibrinogen, fibronectin, vitronectin, immunoglobulins, and Willebrand factor. A key protein for platelet adhesion is fibrinogen [12], while fibronectin, immunoglobulins, and the Willebrand factor show only supportive effects that may be associated with platelet activation [13]. The amount of adsorbed fibrinogen required for platelet adhesion is very small—7 ng/cm [14].

Adsorption of certain procoagulants onto artificial surfaces can activate the intrinsic blood coagulation system, which can lead to the formation of blood clots [10]. Such for example is factor XII, which is auto-activated onto artificial surfaces [1]. Activation of the intrinsic blood coagulation system can also be driven by leukocyte adhesion onto artificial surfaces via tissue factor exposure at the plasma membrane of the bound cells [10]. Some studies have suggested that the initial attachment of platelets to artificial surfaces is increased if blood flow or shear rate is increased. However, platelet attachment depends on the type of material and flow [7]. Kameneva and Antaki [15] described that, when using cardiovascular devices, viscosity is increased at low flow and low shear rates, and erythrocyte aggregation can occur *in vivo* and *in vitro*. Box et al. [16] described that with increasing shear rate, blood viscosity decreases (elevated blood viscosity is known to induce cardiovascular disease). However, the shear rates increase with increasing flow. Westerhof et al. [17] also described that viscosity decreases when the shear rate increases. At high shear rates, the erythrocytes are directed in the direction of flow and the viscosity is lower. At low shear rates, however, erythrocyte aggregation can occur, which increases the viscosity.

Therefore, the aim of the study was to evaluate how different parameters (material, coating, tunnel geometry, length, lumen diameter, and number of lumens) of central venous catheters (CVCs) and flow rates through the catheters affect blood cell damage.

## 2. Methods

### 2.1. Sample Preparation

The study was conducted on 71 CVCs from three different manufacturers. Catheters were addressed as A, B, and C to ensure blind identity, leaving out the name and trademark. We tested 51 single-lumen CVCs from manufacturer A, B, and C (Table 1), and 20 five-luminal CVCs from manufacturer A and B (Table 2). Taking five lumens, blood was only released through the distal lumen.

### 2.2. Blood Samples

Plastic bags containing packed red blood cells (RBC/erythrocyte concentrate) in citrate, phosphate, dextrose, and adenine (CPDA) solution were used as a source of blood. RBC concentrates from four different male donors were obtained; (i) RBC concentration 7.3x10^12^/L, hemoglobin 213 g/L, hematocrit 0.80, and MCV 110 fL; (ii) RCB 7.7x10^12^/L, hemoglobin 205 g/L, hematocrit 0.75, and MCV 98; (iii) RBC 7.2x10^12^/L, hemoglobin 202 g/L, hematocrit 0.74, and MCV 103; (iv) RBC concentration 7.3x10^12^/L, hemoglobin 205 g/L, hematocrit 0.75, and MCV 100 fL.

CVCs were connected to packed red blood bag, and blood was pumped through CVCs and collected into silicone-coated tubes with EDTA to reduce adherence of red cells. Pressure for laminar blood flow through CVC was generated using ECMO (extracorporeal membrane oxygenation) machine pump. Two different flow rates were used, namely, 33 mL/min, which was the lowest possibly generated rate with ECMO pump, and 500 mL/min, which was the highest possibly generated flow rate with the ECMO pump. Utterly different flows were tested to ensure that the flow rates were different enough and could show the impact on blood cell damage.

Damage of blood cells (erythrocytes) was determined by hematological tests (hematocrit, total and free Hb concentration, and hemolysis rate) and by flow cytometry with analysis of microparticles (MPs).

### 2.3. Isolation of Microparticles (MPs)

The whole process was performed at room temperature. The erythrocyte concentrate was centrifuged for 15 min at 2500 g. Thereafter, the plasma was pipetted at about 1 mL above the deposited erythrocytes to avoid sediment. Plasma was centrifuged for 15 min at 2500 g, and 0.5 mL of supernatant was pipetted into microtubes. The density of platelets in plasma was in average 1.4 ± 2.2 × 10^10^/L; MPV was 7.0 ± 3.6 fL. The samples were stored at −80°C for further analyses.

### 2.4. Preparation of Samples for Determination of Number and Concentration of Microparticles and for Labeling with CFSE

Samples were thawed to room temperature and agitated on a vibrating shaker at low rpm (12 rpm). MPs were diluted 1 : 3, then 30 *μ*L of diluted suspension was added to 110 *μ*L of HEPES buffer. The suspension was mixed on a vibrating shaker at low rpm and incubated for 15 min at room temperature in the dark place. After incubation, 4.5 *μ*M CFSE (Life Technologies, CA, USA) was added, shaken on a vibrating shaker at low rpm, and incubated for 15 min at room temperature in the dark. After incubation, 50 *μ*L of polyethylene counting beads (Beckman Coulter, CA, USA) was added and shaken on a vibrating shaker at low rpm. Thus, the sample was prepared for analysis on a flow cytometer.

### 2.5. Sample Preparation and Labeling of Microparticles with CFSE, Annexin V, and CD235a

30 *μ*L of diluted MPs suspension was added to 70 *μ*L of Annexin binding buffer (HEPES), shaken on a vibrating shaker at low rpm, and incubated for 15 min at room temperature in the dark place. After incubation, 4.5 *μ*M CFSE, 10 *μ*L Annexin V (BD Biosciences, Switzerland), and 5 *μ*L CD235a (BD Biosciences, Switzerland) were added, shaken on a vibrating shaker at low rpm, and incubated in the dark for 15 min at room temperature. After incubation, 200 *μ*L of HEPES buffer was added and centrifuged for 30 min at 17,600 g and 20°C. After centrifugation, 200 *μ*L of the supernatant was pipetted and discarded. The sediment (stained MPs) was resuspended with 100 *μ*L of HEPES buffer. Thus, the samples were prepared for analysis on a flow cytometer.

Negative control for Annexin V was prepared with 30 *μ*L of diluted MPs (1 : 3) and 70 *μ*L of CaCl-free buffer, shaken on a vibrating shaker at low rpm, and incubated in the dark for 15 min at room temperature. After incubation, 10 *μ*L of Annexin V was added, shaken on a vibrating shaker at low rpm, and incubated in the dark for 15 min at room temperature. After incubation, 200 *μ*L of CaEP-free HEPES buffer was added, shaken on a vibrating shaker at low rpm, and centrifuged for 30 min at 17,600 g and 20°C. After centrifugation, 200 *μ*L of the supernatant was pipetted and discarded. The sediment was resuspended with 100 *μ*L CaEP-free HEPES buffer and prepared for measurement on a flow cytometer.

### 2.6. Analysis of Microparticles with a Flow Cymometer

**Figure 1:**
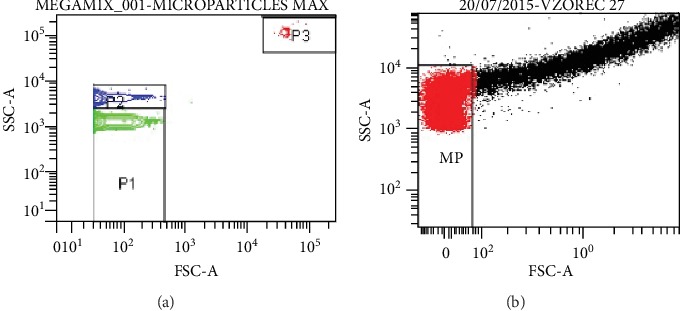
(a) The fluorospheres we separated by size; (b) Point diagram of the MP population.

The flow cytometer was adjusted using a mixture of size-calibrated fluorescent beads (fluorospheres). A pronounced granularity (SSC) was plotted on the *y*-axis and a size (FSC) of the fluorosphere on the *x*-axis (Figure 1(a)). Large-calibrated fluorescent beads allowed the construction of gates for MPs (Figure 1(b)).

**Figure 2:**
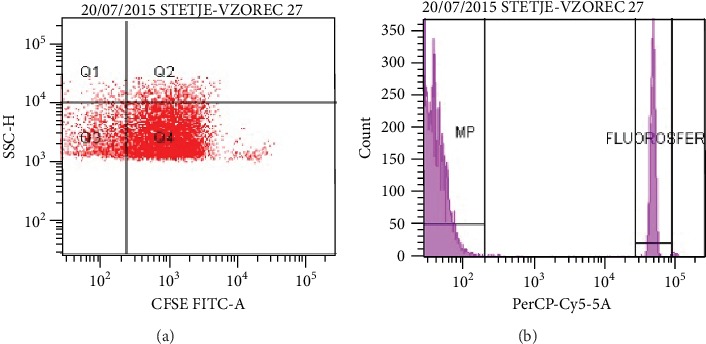
(a) Fluorospheres that allow MPs to be counted; (b) CFSE Positive MPs (Q4).

In the second step, the proportion of all MPs was measured on a flow cytometer. Fluorospheres were added to the sample to allow counting (Figure 2(a)). MPs were labeled with CFSE, with a plotted granularity of MPs (SSC) on the *y*-axis and fluorescence on the *x*-axis (Figure 2(b)).

The concentration of MPs was calculated with the following equation:

(1) $$\begin{array}{c} \text{c}\left(\text{MP}/\text{plasma}\right)=\text{R}\times \frac{N\left(MP\right)}{N\left(FS\right)}\times \frac{V\left(FS\right)}{V\left(MP\right)}\times \text{c}\left(\text{FS}\right), \end{array}$$

where *R* is the dilution factor; *R* = *V* plasma isolated MP/final *V* resuspended MP; *N* (MP) is the number of microparticles measured by a flow cytometer; *N* (FS) is the number of fluorospheres measured by a flow cytometer; *V* (MP) is the sample volume of resuspended microparticles used for analysis on a flow cytometer; *V* (FS) is the volume of added fluorospheres for measurement on a flow cytometer; and *C* (FS) is the original fluorosphere concentration.

The fluorophosphorus concentrations were 0.954x10^6^/mL and 1.005x10^6^/mL.

**Figure 3:**
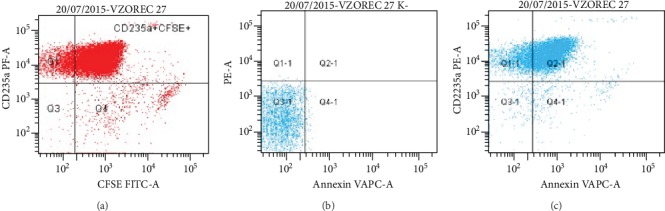
(a) Erythrocyte MPs positive for CD235a and CFSE (Q2); (b) Negative control for Ann V; (c) Population breakdown diagram of MPs on Ann V negative (Q1-1) and positive (Q2-1).

The proportion of erythrocyte MPs labeled with Ann V (APC), CD235a (PE), and CFSE (FITC) was examined on a flow cytometer. Only CFSE-positive MPs were analyzed. MPs that radiated PE fluorescence and were positive for CD235a were plotted on the *y*-axis, and MPs that radiated FITC fluorescence and were positive for CFSE were plotted on the *x*-axis (Figure 3(a)). Negative control for Ann V (Figure 3(b)) was measured to determine the boundary between Ann V positive and negative MPs (Figure 3(c)). MPs were labeled with Ann V in the absence of Ca ions in the negative control. The proportion of erythrocyte MPs positive for CD235a and Ann V was determined (Figure 3(c)).

The erythrocyte MPs concentration was calculated with the following equation:

(2) $$\begin{array}{c} \text{Erythrocyte MP concentration}\left(\text{Ann V}+\text{CD}235\text{a}+\text{CFSE}\right)=\left(\text{concentration of all MP}\times \text{Ann V share}+\text{CD}235\text{a}+\text{CFSE}\right)/100\%. \end{array}$$

### 2.7. Hematological Tests

The rate of hemolysis was calculated with the following equation:

(3) $$\begin{array}{c} \text{Hemolysis }\left(\%\right)=\left(100-\text{hematocrit}\right)\text{ xfree Hb}/\text{total Hb}. \end{array}$$

### 2.8. Hemorheological Calculations

The exposure time of erythrocyte concentrate on CVCs was calculated with the following equation:

(4) $$\begin{array}{c} t=\frac{V}{\Phi }, \end{array}$$

where *V* is the volume of erythrocyte concentrate, and *Φ* is the flow of erythrocyte concentrate.

The mean speed rate of erythrocyte concentrate was calculated with the following equation:

(5) $$\begin{array}{c} v=\frac{\Phi }{S}, \end{array}$$

where *Φ* is the flow of erythrocyte concentrate, and *S* is the cross-sectional area (*S* = *πr*^2^).

The shear rate of the erythrocyte concentrate was calculated with the following equation:

(6) $$\begin{array}{c} \gamma =\frac{v}{h}, \end{array}$$

where *γ* is the shear rate measured in reciprocal seconds (1/s); *h* is the distance between two parallel plates (diameter); and *v* is the medium speed.

The shear stress was calculated with the following equation:

(7) $$\begin{array}{c} \tau =\eta \times \gamma , \end{array}$$

where *η* is the blood viscosity and *γ* is the shear rate.

The dynamic viscosity of the erythrocyte concentrate was calculated with the following Einstein equation:

(8) $$\begin{array}{c} \eta =\eta_{\text{plazme}}\times \left(1+2.5\times \text{Ht}\right), \end{array}$$

where *η*^*plazme*^ is the plasma viscosity, and *η*^*plazme*^ = 1, 5 cP = 1.5 × 10^−3^Pa × s.

## 3. Results

Overall, 71 CVCs were tested with two different flow rates (33 mL/min and 500 mL/min, respectively). Thus, 142 samples were analyzed altogether.

### 3.1. Low Flow Rate (33 mL/min)

There were no statistically significant differences in hematologic parameters and MP concentration (Table 3 and Supplement 1) among CVCs with single lumen. However, there were statistical differences in the hemorheological parameters such as shear rate and shear stress between manufacturer A and B and B and C (*p* < 0.001, respectively), but no differences between manufacturers A and C (Supplement 1).

When testing CVCs with 5 lumens also, no statistically significant differences in MP concentration were found (Table 4 and Supplement 2). A minor difference in hemolysis rate between manufacturer B and A was observed but was not statistically significant (*p* = 0.150). Moreover, there were statistically significant differences in the hemorheological parameters between manufacturers A and B (Supplement 2). The shear rate and shear stress of erythrocyte concentrate were shown significantly higher for manufacturer B than that of manufacturer A (*p* < 0.001, respectively).

Comparison of CVCs with one and five lumens from the same manufacturer also showed differences in some of the parameters (Supplement 3). Manufacturer A showed a higher hemolysis rate with one lumen compared to five lumens (*p* < 0.001). The MP concentration of manufacturer A with one lumen was also statistically higher than that of CVC with five lumens (*p* = 0.015). There were no statistically significant differences in hemorheological parameters between single and five-luminal CVCs from manufacturer A (data not shown). On the other hand, no statistically significant differences in MP concentration were observed when comparing single and five-luminal CVCs from manufacturer B. However, a statistically significant difference in the rate of hemolysis was observed (*p* < 0.001). Similarly as with A also, no differences in hemorrhagic parameters between single and five-luminal CVCs from manufacturer B were observed (data not shown).

### 3.2. High Flow Rate (500 mL/min)

The high flow rate showed no statistically significant differences in MP concentration when comparing CVCs with a single lumen (Table 5 and Supplement 4). However, differences in the concentration of total Hb and the proportion of hematocrit between the catheters were found (Supplement 4). Single-lumen CVCs from manufacturers A, B, and C also exhibited differences in different hemorheological parameters, namely, shear rate and shear stress. The shear rate of erythrocyte concentrate in manufacturer B was statistically higher than that of manufacturer A and C (*p* < 0.001, respectively). There were no statistically significant differences in the shear rate between manufacturers A and C (*p* = 0.290). The shear stress of manufacturer B was significantly higher than that of manufacturer A and C (*p* < 0.001, respectively) and statistically higher for manufacturer C than for manufacturer A (*p* < 0.001).

### 3.3. Comparison of Low and High Flow Rates

Comparison of low and high flow rates showed statistically significant differences in shear rates and shear stress for all three manufacturers (Supplement 5). The high flow rates resulted in a higher shear rate and shear stress. Exposure time was also the parameter that differed among all manufacturers since with low flow rate blood cells were averagely exposed for 9.1 seconds (Table 3) and with a high rate for 0.9 seconds (Table 5). The concentration of MP showed lower concentrations with high flow rates and differed in manufacturer A and C, but not in manufacturer B. On the contrary, it was observed with hematocrit, showing higher levels with high flow rate for all three manufacturers, but only statistically significant for A and C (Supplement 5). The concentration of total Hb with high flow rate was higher for manufacturer A and C (*p* < 0.001, respectively), but lower in manufacturer B. However, the differences were not statistically significant (*p* = 0.790).

## 4. Discussion

The most common complications with CVCs are damage to circulating blood components, the formation of blood clots, hemolysis, and release of hemoglobin into the plasma. In our study, we tested single and five-luminal CVCs from three different manufacturers at two different flow rates of erythrocyte concentrate. Blood cell damage was defined by microparticle and hematologic results. The purpose of the study was to evaluate how different CVC parameters (presence of coating, tunnel geometry, length, lumen diameter, and number of lumens) and flow rate of erythrocyte concentrate through CVC affect blood cell damage.

Microparticles are a potential diagnostic marker and have a specific function in the pathophysiology of the disease. However, the problem is that there is currently no standard method for isolation and analysis of MPs from blood samples. In our study, MPs were isolated by ultracentrifugation of erythrocyte concentrate. MPs were defined as particles smaller than 2 *μ*m expressing phosphatidylserine and glycophorin A on their surface. Expression of phosphatidylserine on the surface of MPs was confirmed by Annexin V labeling, and glycophorin A expression was confirmed by anti-CD235a antibody labeling. MPs were also CFSE positive, meaning that MPs are closed structures—vesicles and can be clearly separated from cell debris. With MP and hematologic analyses, we first identified which CVCs cause more blood cell damage. After these CVC properties and influence of blood flow rate were analyzed.

### 4.1. Impact of Coating

CVCs from manufacturer C had a polyhexanide methacrylate coating; meanwhile, catheters from A and B were uncoated. The low flow rate showed no effect on blood cell damage. However, when comparing CVCs with a high flow rate, statistically significant differences between the manufacturers were observed, namely, the concentration of total Hb and in the proportion of hematocrit. The highest concentration of total Hb and the proportion of hematocrit were observed with CVCs from manufacturer C. Hematocrit is the ratio of erythrocyte volume to the whole blood volume [18]. The higher the proportion of hematocrit, the more erythrocytes remain intact (undamaged), and the higher is the concentration of total Hb. Manufacturer C had a coating, therefore, caused less damage to the blood cells. Catheter coating can play an important role in the traumatic effect of surface. Coated and uncoated CVCs have also been investigated by Chauhan et al. [5], who demonstrated that coated CVCs reduce initial cell adhesion, biofilm formation, and unwanted complications, compared with uncoated CVCs. Krikava et al. [19] proved that polyhexanide-coated CVCs reduce bloodstream infections compared to standard noncoated CVCs. In our study, we observed that a low flow rate had no effect on differences in blood cell damage, as no statistically significant differences in blood cell damage were observed. However, at high flow rates, the coating influenced differences in blood cell damage.

### 4.2. Impact of Length

At a high flow rate, significant differences were observed in the concentration of total Hb and the proportion of hematocrit. The highest total Hb concentration and hematocrit was found for manufacturer C with the longest catheters (20 cm), followed by manufacturer A (with CVCs of 16 sm), and then manufacturer B (catheters of 15 cm). Manufacturer C which had the longest catheters (20 cm) resulted in the least significant damage to blood cells. The most damage was caused by CVCs from manufacturer B having the shortest catheters. In terms of length, it would be expected that the longest catheters cause the most damage to the blood cells since exposure time to artificial surfaces is crucial for cell damage. Ubaldo Vieira Junior et al. [20] found that erythrocyte damage was dependent on exposure time. With longer exposure times, a higher rate of hemolysis and consequently more damage to erythrocytes is expected. It is assumed that the differences in damage to the coating have a greater influence than the length, since CVCs of manufacturer C, which were the longest but also had a coating, caused less damage than CVCs of manufacturer A and B, which were shorter but without coating. It is also assumed that the chemical composition of the catheters has an effect on the differences, but unfortunately, no data were available for our samples. CVCs from manufacturer A were 1 cm longer than CVCs from B, but despite the length and slightly longer exposure time, they caused less damage, which might be attributed to the chemical composition.

### 4.3. Impact of Lumen Diameter

The hemorheological parameters indicate that the surfaces of single and five-luminal CVCs from manufacturer B were the least traumatic since the shear rate and shear stress were statistically different compared to A and C. In hemorrhagic calculations, only the catheter's lumen diameter is considered from the individual CVC properties. Kameneva and Antaki proved [15] that at a low shear rate, erythrocyte aggregation can occur, so it is better that the shear rate is higher. It is also better to have higher shear stress as low shear stress is associated with turbulent currents and cardiovascular complications [21]. When the diameter of the lumen is smaller, the shear rate and the shear stress are higher, meaning the smaller the lumen of the catheter, the less traumatic surface it has.

When comparing single-lumen CVCs with high flow rate differences, the concentration of total Hb and the proportion of hematocrit were observed. CVCs with single lumen from C had a lumen diameter of 1.7 mm, causing the least damage to blood cells, followed by CVCs of manufacturer A, which had a lumen diameter of 1.7 mm, and the most damage was caused by CVCs of manufacturer B, which had a lumen diameter of 1.5 mm. However, hemorrhagic parameters indicate that the surface of CVCs from manufacturer B is typically the least traumatic since the shear rate and shear stress were significantly higher. Depending on the lumen diameter, catheters with a larger diameter are expected to cause more damage as the shear rate and shear stress are lower. In our case, with the larger diameter, lower shear rate, and shear stress, there was less damage to the blood cells. The results are on the contrary when compared to the findings by Kameneva and Antaki [15] and Papaioannou and Stefanadis [21]. The reasons for discrepancies might be variations in CVC coating and the chemical composition which could have a greater influence than the lumen itself, since the CVCs of manufacturer C, which had the largest lumen diameter but had a coating, causing less damage. And CVCs from manufacturer A had the same lumen diameter (1.7 mm).

### 4.4. Impact of Tunnel Geometry

When comparing the distal lumen of the five-luminal CVCs with a low flow rate, only minor differences in the rate of hemolysis were observed, namely, the CVCs of manufacturer B had shown lower hemolysis rate than CVCs from A. However, the hemolysis rate was not statistically significant (*p* = 0.150). Hemolysis is a pathological process that defines erythrocyte damage and is reflected in the release of Hb into the plasma [4, 22]. The higher is the rate of hemolysis, the higher is the concentration of free Hb in plasma, and the greater is the damage to erythrocytes. The results indicate that manufacturer B caused slightly less damage than manufacturer A. Both have different lumen geometries. The distal lumen of CVCs from B had a round shape, while CVCs from A had an irregular shape (Table 2). It is assumed that the CVCs from A might have caused turbulence due to lumen geometry and consequently a slightly increased rate of hemolysis. The use of cardiovascular devices often causes turbulent flows. On the other hand, the blood flow in the human circulatory system is mostly laminar. Kameneva et al. [23] demonstrated that turbulent flows contribute to an increased rate of hemolysis. Artificial surfaces must be smooth, hemocompatible, and without sharp edges to reduce the probability of turbulent flows [3]. However, it cannot be argued that the geometry had an effect on differences in blood cell damage since the differences were not statistically significant. Single-lumen catheters from manufacturer A are also round shape, but the free Hb concentration, hemolysis rate, concentration of MPs, and erythrocyte MPs were statistically higher than for five-luminal catheters whose distal lumen was not round. Given the geometry of the lumen, one would expect that five-luminal CVCs would do more damage than a single-lumen CVCs. In this case, the number of lumens is assumed to have a greater effect on the difference in damage than the geometry of the tunnel. Single-lumen CVCs of manufacturer B have the same lumen geometry as the distal five-luminal CVCs, so the geometry in this case also had no effect on the differences in blood cell damage.

### 4.5. Impact of Lumen Number

Single-lumen catheters of manufacturer A showed higher concentrations of free Hb, hemolysis rates, total, and erythrocyte MPs compared to five-luminal catheters. Mesri and Altieri [24] demonstrated that the number of MPs doubles during different inflammatory conditions. Inflammation is part of a nonspecific immune response that occurs in response to any type of personal injury [25]. The same was observed in single-lumen catheters of manufacturer B, which showed higher concentrations of free Hb and hemolysis rate compared to the five-luminal catheters. However, there were no differences in the concentration of MPs. In both manufacturers, five-luminal CVCs produced statistically significantly less blood cell damage compared to one-lumen CVCs. The distal lumen in catheters with five lumens is surrounded by the proximal lumen and the three median lumens. The diameter of a five-luminal catheter (the diameter of all five lumens together) is about 5 times larger than that of a single lumen. Five-luminal CVCs are thus more stable because they are thicker and harder to bend. As a result, five-luminal catheters have fewer mechanical occlusions that cause damage to the blood cells. Catheter occlusion is defined as partial or complete obstruction. Obstruction can occur inside or outside the human circulatory system when using CVCs. Internal obstruction may result from incorrect insertion: e.g., the catheter may bend or the tip of the catheter leans against the wall of the vessel instead of floating freely in the vessel. External obstruction involves catheter twisting [26, 27]. The number of lumens affects differences in erythrocyte damage. In catheters with multiple lumens, there was statistically significant less damage to the blood cells.

### 4.6. Impact of Flow Rate

CVCs of manufacturers A and C caused more blood cell damage with a low flow rate than with high flow rate since the Hb concentration and hematocrit at low flow rate were lower. Meanwhile, the concentration of total and erythrocyte MPs was significantly higher compared to the high flow rate. Minor differences in the concentration of erythrocyte MPs were observed between single-lumen catheters of manufacturer B, with slightly higher erythrocyte MPs concentrations at a low flow rate than at high flow rates. However, the difference was not statistically significant (*p* = 0.190). All three manufacturers showed lower shear rates and shear stress with low flow rates and significantly longer exposure time. Ubaldo Vieira Junior et al. [20] found that erythrocyte damage was dependent on exposure time, and lower hemolysis rates are expected at low exposure times. We also demonstrated that exposure times at high flow rates were lower and that there was less blood cell damage with manufacturer A and C. There were no statistically significant differences in blood cell damage with manufacturer B, but the results indicated that there is less damage with high flow rates. At a high shear rate, erythrocytes are directed in the flow direction [17], whereas, at a low shear rate, erythrocyte aggregation can occur [15], so it is preferable that the shear rate is higher [20]. It is also better to have higher shear stress as low shear stress is associated with turbulent flows [21], which contribute to an increased rate of hemolysis [17]. The relationship between low shear stress and cardiovascular complications has also been reported by Jeong and Rosenson [28] and Carallo et al. [29]. In our case, the shear rate and shear stress were lower with low flow rate resulting in more blood cell damage.

In conclusion, catheter characteristics did not influence blood cell damage with low flow rate but had an effect with high flow rate. The coating at high flow rate influenced the hematological parameters, so CVCs from manufacturer C had a polyhexanide methacrylate coating which might have caused less blood cell damage than noncoating CVCs from manufacturers A and B. The lumen length, diameter, or tunnel geometry did not exactly influence the formation of MPs, although differences in hematological parameters were observed. We believe that the coating and chemical composition of the catheter had a much greater effect on the differences in blood cell damage than the length or diameter of the catheter. The number of lumens was predicted to have a greater effect on the differences in erythrocyte damage than the tunnel geometry, which revealed that the number of lumens influenced the formation of MPs and hematological parameters. CVCs from manufacturers A and B that had five lumens thus caused less damage to blood cells than CVCs with single lumen. In addition to the characteristics of catheters, different blood flow rates were tested and proved that a high flow rate of 500 mL/min caused significantly less blood cell damage than the low flow rate of 33 mL/min. Utterly different flows were tested to ensure that the flow rates were different enough and could show the impact on blood cell damage. CVC dysfunction is a frequent problem and often is defined as a blood flow <300 mL/min (<100–350 mL/min). In one study, practice change from one to another CVC resulted in the routine achievement of blood flow rates of 500 mL/min. Previously, 300-400 mL/min blood rate was the norm [30]. *In vivo* different flow rates for transfusing or withdrawing blood may apply, depends on the usage and manufacturer specifics. One of the manufacturers in our study recommended in 14G distal lumen a maximal flow of 6500 mL/h (108 mL/min), the other 3780 mL/h (63 mL/min) for the same gauge size. Taking all that, a better CVC design produces less MPs, less apoptotic erythrocytes, and less hemolysis. The increased flow rate was achieved without any apparent increase in venous pressure, but by the possibility that increased recirculation was occurring. Thus, using coated, more-luminal CVCs with the high flow rates could be an applicable practical prove for less blood cell damage and could enhance the efficacy of CVC usage. Identification of such parameters might offer the potential to improve efficiency of targeted engagements and reliable blood delivery.

With the inclusion of different CVCs, it has been showed that the selection of catheters has different effects on red blood damage. In order to eliminate the bias of the conducted analysis, CVCs from different manufacturers were included in the study. However, catheters from the same manufacturer, with and without the relevant characteristics, would have to be tested for much more accurate evaluations on the impact of characteristics, which was not the case in our study. Properties of CVCs are important factors for quality patient care, especially when transfusing blood with high flow rates as we want to provide high-quality blood with as few damaged cells as possible.

## Figures and Tables

**Table 1:** Characteristics of CVCs with single lumen from manufacturers A, B, and C.

	A (*N* = 17)	B (*N* = 17)	C (*N* = 17)
Material	Polyurethane	Polyurethane	Polyurethane
Coating	/	/	Polyhexanide methacrylate
Length	16 cm	15 cm	20 cm
Luminal diameter	1.7 mm	1.5 mm	1.7 mm
Geometry	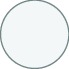	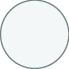	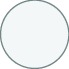

**Table 2:** Characteristics of CVCs with 5 lumens from manufacturers A and B.

	A (*N* = 10)	B (*N* = 10)
Material	Polyurethane	Polyurethane
Coating	/	/
Length	16 cm	15 cm
Distal lumen diameter	1.7 mm	1.5 mm
Geometry	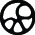	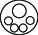

**Table 3:** Measurements from single-lumen CVCs after 33 mL/min flow rate of erythrocyte concentrate.

	A (*N* = 17)		B (*N* = 17)		C (*N* = 17)	
	Mean	SE	Mean	SE	Mean	SE
Total Hb (g/L)	195.7	1.96	200.0	4.46	203.3	3.83
Free Hb (g/L)	5.4	0.44	5.9	0.5	5.9	0.5
Hematocrit (%)	0.68	0.01	0.69	0.01	0.7	0.01
Hemolysis (%)	2.75	0.22	2.88	0.2	2.87	0.23
Microparticles (MP/mL)	557,629	89,550	553,899	120,653	588,968	114,092
Erythrocyte MP (MP/mL)	270,462	46,708	288,358	80,665	322,014	71,361
Medium speed (cm/s)	6.064	0	7.79	0.00002	6.064	0
Shear rate (1/s)	35.67	0	51.936	0.00012	35.67	0
Shear stress (Pa)	0.144	0.0009	0.212	0.0027	0.147	0.0016
Exposure time (s)	9.09	0	9.09	0	9.09	0
Dynamic viscosity (Pa·s)	0.00403	0.00003	0.00408	0.00005	0.00413	0.00005

**Table 4:** Measurements from five-luminal CVCs after 33 mL/min flow rate of erythrocyte concentrate.

	A (*N* = 10)		B (*N* = 10)	
	Mean	SE	Mean	SE
Total Hb (g/L)	194.4	3.44	200.0	4.75
Free Hb (g/L)	2.4	0.16	2.0	0.37
Hematocrit (%)	0.69	0.01	0.7	0.01
Hemolysis (%)	1.23	0.08	0.98	0.02
Microparticles (MP/mL)	198,430	78,799	256,958	132,521
Erythrocyte MP (MP/mL)	85,398	32,539	79,606	32,676
Medium speed (cm/s)	6.064	0.00039	7.79	0
Shear rate (1/s)	35.67	0.0023	51.94	0.0004
Shear stress (Pa)	0.145	0.0015	0.214	0.0029

**Table 5:** Measurements from single-lumen CVCs after 500 mL/min flow rate of erythrocyte concentrate.

	A (*N* = 17)		B (*N* = 17)		C (*N* = 17)	
	Mean	SE	Mean	SE	Mean	SE
Total Hb (g/L)	216.5	0.85	198.45	3.71	222.86	1.51
Hematocrit (%)	0.75	0	0.7	0.01	0.78	0.01
Microparticles (MP/mL)	268,723	54,641	434,352	83,019	257,923	35,026
Erythrocyte MP (MP/mL)	110,036	21,764	173,025	33,484	111,177	18,920
Medium speed (cm/s)	91.84	0	117.99	0	91.84	0
Shear rate (1/s)	540.24	0	786.59	0	540.24	0
Shear stress (Pa)	2.33	0.008	3.24	0.038	2.39	0.011
Exposure time (s)	0.6	0	0.6	0	0.6	0
Dynamic viscosity (Pa·s)	0.00432	0.000014	0.00412	0.000048	0.00443	0.000021

## Data Availability

The data are confidential.

## Supplementary Materials

Supplementary Materials Supplement 1: statistical analysis with *p* values for comparisons between single-lumen CVCs after 33 mL/min flow rate of erythrocyte concentrate. Supplement 2: statistical analysis with *p* values for comparisons between five-luminal CVCs after 33 mL/min flow rate of erythrocyte concentrate. Supplement 3: statistical analysis with *p* values for comparisons between single-lumen and five-luminal CVCs from the same manufacturer after 33 mL/min flow rate of erythrocyte concentrate. Supplement 4: statistical analysis with *p* values for comparisons between single-lumen CVCs after 500 mL/min flow rate of erythrocyte concentrate. Supplement 5: statistical analysis with *p* values for comparisons between low flow rate of 33 mL/min and high flow rate of 500 mL/min for single-lumen CVCs.
