# Supplementary material for: An In Vitro Evaluation of the Red Cell Damage and Hemocompatibility of Different Central Venous Catheters
Source: Biomed Res Int. 2020 Apr 14;2020:8750150. doi: 10.1155/2020/8750150 (PMC7178527; doi:10.1155/2020/8750150)
Supplement: Supplementary Materials — Supplement 1: statistical analysis with p values for comparisons between single-lumen CVCs after 33 mL/min flow rate of erythrocyte concentrate. Supplement 2: statistical analysis with p values for comparisons between five-luminal CVCs after 33 mL/min flow rate of erythrocyte concentrate. Supplement 3: statistical analysis with p values for comparisons between single-lumen and five-luminal CVCs from the same manufacturer after 33 mL/min flow rate of erythrocyte concentrate. Supplement 4: statistical analysis with p values for comparisons between single-lumen CVCs after 500 mL/min flow rate of erythrocyte concentrate. Supplement 5: statistical analysis with p values for comparisons between low flow rate of 33 mL/min and high flow rate of 500 mL/min for single-lumen CVCs. [file 8750150.f1.docx]

A determination of red blood cell damage as a novel method for functional characterisation of central venous catheters: a pilot study

David Stubljar, Stefan Grosek, Alojz Ihan

**Supplemental tables**

Supplement 1: Statistical analysis with p-values for comparisons between single lumen CVCs after 33 mL/min flow rate of erythrocyte concentrate

|  | **A - B** | **A - C** | **B - C** |
| --- | --- | --- | --- |
|  | p-value | p-value | p-value |
| **Total Hb** | 0.380 | 0.090 | 0.580 |
| **Free Hb** | 0.450 | 0.450 | 1.000 |
| **Hematocrit** | 0.410 | 0.070 | 0.490 |
| **Hemolysis** | 0.670 | 0.720 | 0.970 |
| **Microparticles** | 0.980 | 0.830 | 0.830 |
| **Erythrocyte MP** | 0.850 | 0.550 | 0.760 |
| **Shear rate** | <0.001 | 0.320 | <0.001 |
| **Shear stress** | <0.001 | 0.710 | <0.001 |

Supplement 2: Statistical analysis with p-values for comparisons between five-luminal CVCs after 33 mL/min flow rate of erythrocyte concentrate

|  | **A - B** |
| --- | --- |
|  | p-value |
| **Total Hb** | 0.350 |
| **Free Hb** | 0.270 |
| **Hematocrit** | 0.650 |
| **Hemolysis** | 0.150 |
| **Microparticles** | 0.690 |
| **Erythrocyte MP** | 0.910 |
| **Shear rate** | <0.001 |
| **Shear stress** | <0.001 |

Supplement 3: Statistical analysis with p-values for comparisons between single lumen and five-luminal CVCs from the same manufacturer after 33 mL/min flow rate of erythrocyte concentrate

|  | **A1 - A5** | **B1 - B5** |
| --- | --- | --- |
|  | p-value | p-value |
| **Total Hb** | 0.730 | 1.000 |
| **Free Hb** | <0.001 | <0.001 |
| **Hematocrit** | 0.310 | 0.750 |
| **Hemolysis** | <0.001 | <0.001 |
| **Microparticles** | 0.015 | 0.220 |
| **Erythrocyte MP** | 0.014 | 0.180 |

Supplement 4: Statistical analysis with p-values for comparisons between single lumen CVCs after 500 mL/min flow rate of erythrocyte concentrate

|  | **A - B** | **A - C** | **B - C** |
| --- | --- | --- | --- |
|  | p-value | p-value | p-value |
| **Total Hb** | <0.001 | 0.001 | <0.001 |
| **Hematocrit** | <0.001 | <0.001 | <0.001 |
| **Microparticles** | 0.110 | 0.870 | 0.053 |
| **Erythrocyte MP** | 0.130 | 0.970 | 0.110 |
| **Shear rate** | <0.001 | 0.290 | <0.001 |
| **Shear stress** | <0.001 | <0.001 | <0.001 |

Supplement 5: Statistical analysis with p-values for comparisons between low flow rate of 33 mL/min and high flow rate of 500 mL/min for single lumen CVCs

|  | **A↓ - A↑** | **B↓ - B↑** | **C↓ - C↑** |
| --- | --- | --- | --- |
|  | p-value | p-value | p-value |
| **Total Hb** | <0.001 | 0.790 | <0.001 |
| **Hematocrit** | <0.001 | 0.580 | <0.001 |
| **Microparticles** | 0.011 | 0.420 | 0.007 |
| **Erythrocyte MP** | 0.005 | 0.190 | <0.001 |
| **Shear rate** | <0.001 | <0.001 | <0.001 |
| **Shear stress** | <0.001 | <0.001 | <0.001 |
| **Exposure time** | <0.001 | <0.001 | <0.001 |
